# Sustainable Congo red (CR) and hexavalent chromium [Cr(vi)] removal using graphene functionalized aminated lignin composite: mechanistic and reusability insights

**DOI:** 10.1039/d5ra08641h

**Published:** 2026-03-16

**Authors:** Md. Masum Billah, S. M. Fazle Rabbi, Md. Kamruzzaman, Mohammad Amirul Hoque, Riyadh Hossen Bhuiyan, Israt Jahan Nisa

**Affiliations:** a Department of Applied Chemistry and Chemical Engineering, Gopalganj Science and Technology University Gopalganj 8105 Dhaka Bangladesh mkamruzzamandu81@gmail.com; b Fiber and Polymer Research Division, BCSIR Dhaka Laboratories, Bangladesh Council of Scientific and Industrial Research Bangladesh

## Abstract

This research aims at introducing a new composite capable of removing both a textile dye [Congo red (CR)] and a toxic metal, [hexavalent chromium Cr(vi)], from wastewater using a single material. The composite was developed through a facile and eco-friendly approach utilizing microwave-exfoliated graphene (MG) with bio-derived aminated lignin (AL). Unlike most studies that investigate either dyes or heavy metals separately, this work demonstrates the dual functionality of MG–AL in targeting both classes of contaminants within one framework, while also providing kinetic, isotherm, and thermodynamic insights. The synergistic combination of MG and AL, not previously reported, enhanced adsorption selectivity, capacity, and stability by preventing graphene aggregation and improving pollutant affinity through amine functionalization. Comprehensive characterization using FTIR, SEM, TGA, and XRD confirmed successful functionalization, enhanced stability, and improved crystallinity. Under optimal conditions, the composite achieved remarkable maximum adsorption capacities of 121.3 mg g^−1^ for CR and 196.4 mg g^−1^ for Cr(vi), surpassing many reported biomass-based adsorbents. Adsorption followed pseudo-second-order kinetics and Langmuir isotherm, indicating chemisorption and monolayer coverage. Thermodynamic parameters confirmed that the adsorption process was spontaneous (Δ*G*^0^ = −9.09 kJ mol^−1^ for CR dye, and Δ*G*^0^ = −14.15 kJ mol^−1^) and endothermic (Δ*H*^0^ = 53.02 kJ mol^−1^ for CR dye, and Δ*H*^0^ = 49.08 kJ mol^−1^ for Cr(vi)). Notably, the composite retained significant removal efficiency after five regeneration cycles [80.89% for CR dye and 85.31% for Cr(vi)], outperforming many graphene or lignin-based adsorbents typically tested for fewer cycles. Mechanistic studies indicate that electrostatic attraction between protonated amine groups of AL and anionic pollutants (HCrO_4_^−^/Cr_2_O_7_^2−^ and dye sulfonate groups) plays the predominant role in adsorption, supplemented by π–π interactions with defect-rich graphene surfaces and hydrogen bonding between functional groups. This dual-function, reusable adsorbent advances lignin valorization and aligns with green chemistry principles, providing a scalable and versatile material for wastewater treatment.

## Abstract 2

A sustainable graphene–aminated lignin (MG–AL) composite achieves dual removal of Cr(vi) and Congo Red from wastewater *via* synergistic amine functionalization, defect-rich graphene, high chemisorption, and excellent reusability.

## Graphical abstract

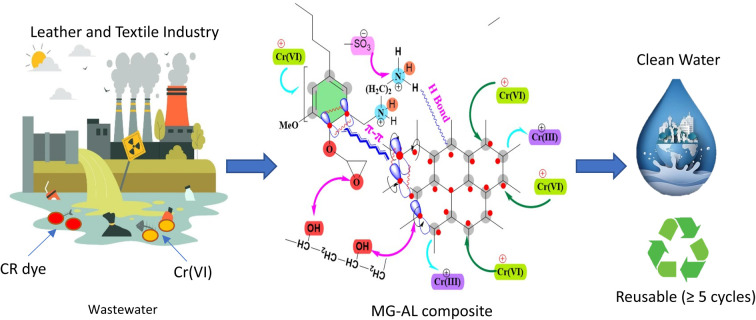

## 1. Introduction

The rapid growth of industrial sectors such as textiles, paper, leather, dyeing, and metal processing has resulted in the substantial discharge of synthetic dyes and heavy metal ions, most notably Congo red (CR) and hexavalent chromium [Cr(vi)], into aquatic environments, posing significant ecological and human health hazards.^1,2^ Azo dyes like CR are of particular concern due to their complex aromatic structures, high water solubility, and strong water resistance to biodegradation, making them persistent and challenging contaminants in wastewater.^3,4^ When these dyes enter aquatic environments, they not only cause vivid coloration but also obstruct light penetration, interfere with photosynthesis, and can generate carcinogenic or mutagenic byproducts through microbial or photochemical reactions.^5^ Similarly, Cr(vi), a highly toxic, mobile heavy metal and persistent environmental pollutant, is widely recognized for its strong oxidizing properties, high solubility, non-biodegradability, and severe health risks, including carcinogenicity, mutagenicity, and cellular toxicity even at trace concentrations.^6^ In aquatic environments, chromium primarily exists as Cr(iii) and Cr(vi); while Cr(iii) is an essential micronutrient, Cr(vi) poses a significant threat due to its ease of absorption by living organisms.^7^ Its ionic forms, hydrogen chromate (HCrO_4_^−^), chromate (CrO_4_^2−^), and dichromate (Cr_2_O_7_^2−^), readily diffuse through biological membranes, causing extensive biochemical and genetic damage.^8,9^ The alarming mobility, bioavailability, and toxicity of Cr(vi) make it one of the most dangerous and persistent contaminants in aquatic systems, necessitating urgent and efficient removal strategies.^10^ Conventional wastewater treatment methods, such as coagulation, chemical oxidation, and biological degradation, often suffer from inherent drawbacks such as incomplete removal, secondary pollution, high operational costs, and limited applicability to complex effluents. In this context, adsorption has emerged as one of the most effective and versatile techniques due to its simplicity, high removal efficiency, operational flexibility, and economic viability.^11,12^ Despite the effectiveness of commercial adsorbents such as activated carbon, their practical application is often limited by high regeneration costs, limited reusability, and environmental concerns associated with their production and disposal.^13^ This has led to a search for sustainable, affordable, and high-performance alternative adsorbents, particularly those sourced from renewable biomasses.

Graphene, a two-dimensional sheet of sp^2^-hybridized carbon atoms arranged in a hexagonal lattice, exhibits exceptional properties including an ultra-high specific surface area, excellent chemical stability, superior electron mobility, and outstanding adsorption capacity. However, its practical application in water remediation is limited by low selectivity, a tendency to aggregate due to van der Waals forces, hydrophobic properties, and high production expenses.^14^ These challenges limit its efficiency and scalability in practical environmental applications. Lignin, a plentiful byproduct from the pulp and paper sector, has recently attracted interest as a sustainable and carbon-rich resource for creating advanced materials. Although lignin inherently contains aromatic rings and various functional groups that can interact with dye and metal molecules, its unmodified form often suffers from low adsorption performance due to limited surface area, inadequate porosity, and poor structural accessibility.^15^ To address these challenges, strategies involving chemical modification by introducing amine groups into lignin through amination improve electrostatic interactions with anionic dyes like CR, thereby synergistically increasing overall adsorption efficiency.^16^ However, its naturally low surface area and structural instability restrict its independent adsorption capacity. A composite of graphene and aminated lignin (MG–AL) is developed to overcome these challenges. This hybrid combines graphene's extensive surface area and π–π interaction capability with the selective, hydrophilic, and biodegradable properties of aminated lignin. The amine groups not only boost pollutant affinity but also stabilize graphene sheets in water, preventing aggregation.^17,18^ This synergistic approach improves adsorption efficiency, selectivity, and environmental compatibility, effectively overcoming the main limitations of the individual components and providing a viable, scalable solution for dye removal applications.^19^

In recent years, a wide range of biomass-derived adsorbents has been investigated for the efficient removal of CR dye and Cr(vi), and other synthetic dyes and heavy metals from aqueous solutions. For instance, Zourou *et al.* developed a graphene oxide- CuFe_2_O_4_ nanohybrid *via* solvothermal self-assembly, which exhibited an adsorption capacity ranging from 15.94 to 376.97 mg g^−1^ for CR (initial concentration: 20–50 mg L^−1^; contact time: 420 min). It has a lower capacity but offers easier separation.^20^ Similarly, Chu *et al.* fabricated lignin/graphene aerogels, further functionalized as LGA@PEI and LGA@PDA, which demonstrated impressive Cr(vi) adsorption capacities of 209.85 ± 3.25 mg g^−1^ and 240.39 ± 3.26 mg g^−1^, respectively, highlighting the potential of lignin-graphene aerogels for heavy metal remediation.^21^ Despite these advances, several challenges remain unresolved: (i) most studies address either dye removal or heavy-metal adsorption individually, while real industrial effluent typically contains both contaminants simultaneously; (ii) the synergistic integration of AL with MG has not been reported, leaving unexplored opportunities for combining lignin's functional groups with graphene's high surface area; (iii) many reports provide adsorption data but lack a comprehensive mechanistic framework that integrates kinetics, isotherms, thermodynamics, and regeneration; (iv) reusability is often restricted to two or three cycles, with sharp efficiency decline beyond that, limiting real-world applicability; (v) structural–performance correlations, such as how functionalization and morphology affect pollutant affinity, remain insufficiently studied; and (vi) very few studies benchmark their adsorbents against recent high-performance systems or assess sustainability and scalability for industrial treatment.

**Fig. 1:**
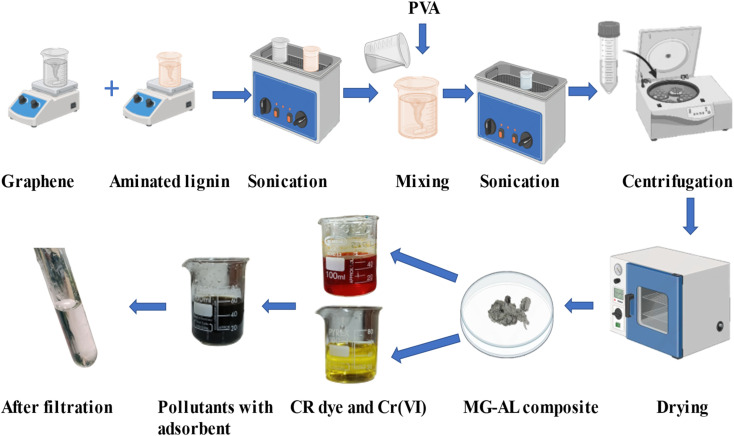
Graphical abstract of microwave graphene–aminated lignin (MG–AL) composite synthesis.

This study addresses these critical gaps by synthesizing MG–AL composite, where amine functional groups enhance electrostatic affinity toward CR dye and Cr(vi), while graphene improves dispersion, stability, and structural integrity of the lignin matrix (follow the graphical abstract shown in Fig. 1). The composite exhibits dual adsorption capability, achieving high capacities, surpassing many biomass and graphene-based adsorbents reported to data.^22^ Unlike most earlier works, adsorption performance is systematically explained through integrated kinetic, isotherm, and thermodynamic modeling, supported by mechanistic insights such as electrostatic attraction, π–π stacking, hydrogen bonding, and possible partial reduction of Cr(vi).^23^ Importantly, the material retains significant efficiency after five consecutive regeneration cycles, demonstrating superior reusability compared to conventional systems. By valorizing lignin an abundant agro-industrial byproduct into a high-value composite using an eco-friendly synthesis, this work not only advances the field of multifunctional bio-nanocomposites but also contributes a scalable, sustainable solution that aligns with green chemistry and circular bioeconomy principles, offering practical potential for wastewater treatment.

Therefore, the present study aims to develop a multifunctional and sustainable adsorbent by synthesizing the MG–AL composite and to systematically evaluate its ability to remove both CR dye and Cr(vi) from aqueous media. The work focuses on (i) synthesizing and characterizing the MG–AL hybrid to confirm structural and functional integration; (ii) evaluating its dual-adsorption performance under key operational parameters; (iii) elucidating adsorption mechanisms through kinetic, isotherm, thermodynamic, and reduction analyses; and (iv) assessing its regeneration stability over multiple cycles. These objectives collectively seek to demonstrate a scalable, high-efficiency, and reusable bio-nanocomposite that addresses the limitations of existing single-pollutant adsorbents and advances wastewater treatment.

## 2. Experimental

### 2.1 Materials

Graphite powder was collected from Loba Chemie, India (98% pure), and lignin was prepared from coconut husk. The rest of the chemicals used were as follows-ammonium bicarbonate (Fine Chem Industries, Mumbai), dimethyl sulfoxide (Merck, Germany), epichlorohydrin (Fisher scientific, USA), ethylenediamine (Merck, Germany), ethanol (Sigma-Aldrich, Poland), polyvinyl alcohol (Loba Chemie, India), dimethyl formamide (Sigma-Aldrich, Poland), Congo red dye (Fine Chem Industries, Mumbai), potassium dichromate (Sisco Research Laboratories, India), and deionized water. All the chemicals were of analytical grade with high purity.

### 2.2 Instrumentation

A roller ball mill (Locally manufactured) was used to achieve uniform mixing of the materials through high-energy collisions and friction. A vacuum oven (Daihan Labtech LVO-2030) was employed for low-temperature drying of samples under reduced pressure (50 mmHg). A microwave oven (LG MS1947C/01) was utilized to provide rapid and uniform radiation for microwave exfoliation. A muffle furnace (Carbolite, CWF 1200) was used, ensuring a clean and controlled environment free from combustion gases for high-temperature processing such as calcination and thermal decomposition of residue. Ultrasonication was carried out using a Bandelin DT 156 BH (Germany) operating at 35 kHz and 180 W, which was employed to facilitate uniform dispersion and interaction of components during composite synthesis under controlled temperature conditions. Centrifugation was performed using a Sigma 2-16P (Germany) at 8000 rpm for 10 minutes to effectively separate the synthesized composite material from the dispersion without compromising its structural integrity.

#### 2.2.1 Synthesis of microwave graphene (MG)

MG was synthesized using a mechanochemical method,^20^ where graphite powder and ammonium bicarbonate were combined in a 1 : 2 weight ratio and subjected to ball milling for 2 hours at a rotational speed of 120 rpm to ensure uniform mixing. The resulting mixture was then preheated in a vacuum oven at 60 °C for 20 minutes. Subsequently, the pretreated material was exposed to microwave irradiation at 2450 MHz and 800 W for 120 seconds. During this process, the thermal decomposition of ammonium bicarbonate released gaseous byproducts (CO_2_, NH_3_, and H_2_O), generating high interlayer pressure that facilitated the rapid exfoliation of graphite into graphene. After exfoliation, the residual byproducts were removed through thermal annealing in a muffle furnace at 500 °C for 5 minutes.^24^

#### 2.2.2 Synthesis of aminated lignin (AL)

AL was synthesized through a two-step functionalization process. Initially, 5.0 g of alkali lignin was dissolved in 100 ml of dimethyl sulfoxide (DMSO) under continuous stirring at 60 °C to obtain a homogeneous solution. Subsequently, 1.5 g of epichlorohydrin was added dropwise, and the reaction mixture was maintained at 70 °C for 3 hours to allow for the activation of lignin through epoxidation. Following this, 5.0 g of ethylenediamine (EDA) was gradually introduced, and the amination reaction proceeded at 80 °C under reflux conditions for an additional 6 hours. Upon completion, the mixture was cooled to room temperature and slowly poured into 300 mL of cold ethanol with vigorous stirring to induce precipitation. The resulting solid was collected by filtration, thoroughly washed with ethanol and deionized water to remove unreacted amines and byproducts, and then dried in a vacuum oven at 50 °C for 12 hours to yield the final AL product.^25^

#### 2.2.3 Synthesis of MG–AL composite

To fabricate a composite material, 0.7 g of MG was dispersed in 20 ml of DMF and stirred for 30 minutes. Similarly, 0.3 g of AL was dissolved in 20 ml of DMF and stirred for the same duration. Both dispersions were subsequently subjected to ultrasonication for 30 min to enhance dispersion and stability. The two solutions were then combined, and 1–2 wt% of PVA was added as a binding agent. The resulting mixture was stirred and further sonicated for 30 min to ensure homogeneity and complete incorporation of PVA. The final dispersion was centrifuged and washed to remove any undispersed residues, and the obtained composite was dried in a vacuum oven at 60 °C to eliminate residual solvent.^26^
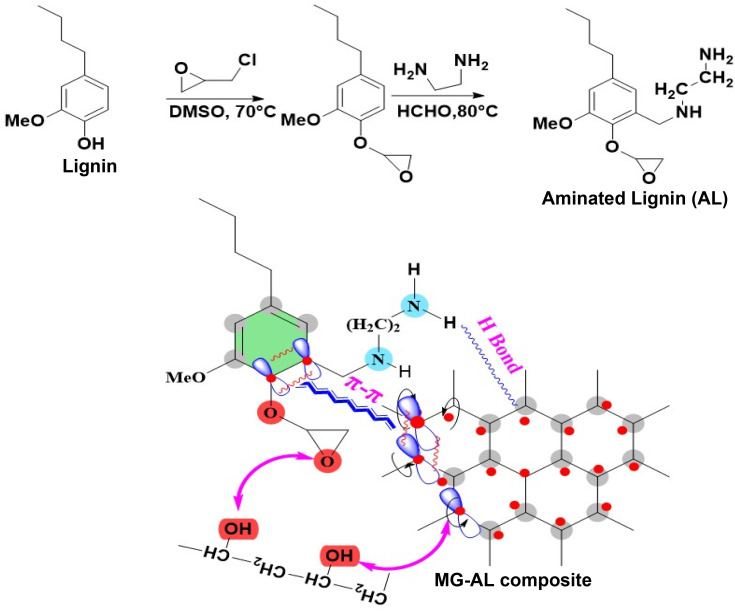


#### 2.2.4 Characterization

Characterization of the synthesized materials was carried out using several analytical techniques. Fourier transform infrared (FT-IR) spectroscopy (Frontier, PerkinElmer, UK) was performed in transmittance mode with 16 scans per sample, using KBr pellets (3 mg sample: 250 mg KBr), across a spectral range of 4000–500 cm^−1^ at a resolution of 4 cm^−1^. Thermogravimetric analysis (TGA) was conducted on a PerkinElmer analyzer under a nitrogen atmosphere (22 mL min^−1^), where approximately 5 mg of sample was heated from room temperature to 800 °C at a rate of 10 °C min^−1^ to assess weight loss and thermal stability. Surface morphology was examined *via* scanning electron microscopy (SEM; EV018, Carl Zeiss, Germany) after platinum sputter-coating the samples. X-ray diffraction (XRD) analysis was carried out using a PerkinElmer diffractometer with Cu-Kα radiation (*λ* = 1.5406 Å) over a 2*θ* range of 5–80°, at a scan rate of 0.05° s^−1^ and a step size of 0.02°, operating at 40 kV and 30 mA, to determine crystallographic structure and interlayer spacing.

#### 2.2.5 Adsorption analysis

Batch adsorption experiments were carried out to evaluate the performance of the MG–AL composite for the removal of CR dye and Cr(vi) under varying experimental conditions. The effects of initial pollutant concentration (20–120 mg L^−1^), pH (1–12), contact time (0–140 min), and adsorbent dosage (0.02–0.1 g) were systematically investigated at a constant agitation speed of 300 rpm and room temperature 9303 K). Unless otherwise stated, all adsorption experiments were conducted under optimized conditions, pH 4 for CR dye and pH 2 for Cr(vi), using an adsorbent dose of 0.05 g, which ensured maximum adsorption performance and reliable comparison across experiments. For CR experiments, UV-Vis spectroscopy (PerkinElmer Lambda 35) was used to determine residual dye concentration, while Cr(vi) concentration was quantified using atomic absorption spectroscopy (NovAA 400 P, Germany). The adsorption capacity (*Q*_e_) and removal efficiency were calculated as:1
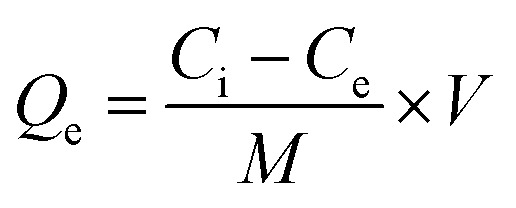
2
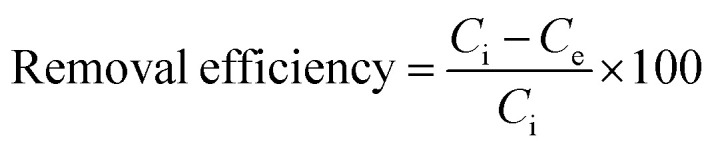
where *C*_i_ and *C*_e_ (mg L^−1^) are the initial and final concentration of CR and Cr(vi), *V* (L) is the solution volume, and *M* (g) is the mass of the adsorbent.^27^ All experiments were carried out in triplicate, and average values are reported to ensure accuracy and reproducibility.

#### 2.2.6 Thermodynamic study

The thermodynamic behavior of CR dye and Cr(vi) adsorption onto the MG–AL composite was evaluated at different temperatures (303, 308, and 313 K) under optimized experimental conditions. The distribution coefficient (*K*_c_) was calculated according to eqn (3):3
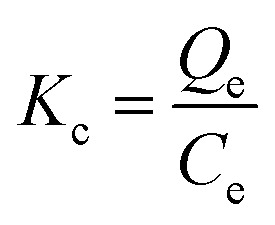
where *Q*_e_ (mg g^−1^) is the equilibrium adsorption capacity and *C*_e_ (mg L^−1^) is the equilibrium concentration of the adsorbate in solution.^28^

#### 2.2.7 Adsorption kinetics

To elucidate the adsorption mechanism of CR dye and Cr(vi) onto the MG–AL composite, kinetic data were analyzed using pseudo-first-order, pseudo-second-order, and intra-particle diffusion models. The linearized form of these models is expressed as follows:

Pseudo-first-order kinetic model

4 ln(*Q*_e_ − *Q*_*t*_) = ln *Q*_e_ − *k*_1_*t*

Pseudo-second-order kinetic model 5
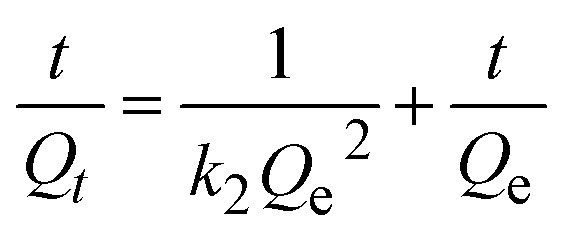


Intra-particle diffusion model

6 *Q*_*t*_ = *k*_diff_*t*^1/2^ + *C*

where *Q*_*t*_ and *Q*_e_ (mg g^−1^) denote the amounts of CR and Cr(vi) adsorbed at time *t* (min) and equilibrium, respectively. *k*_1_ (min^−1^), *k*_2_ (g mg^−1^ min^−1^), and *k*_diff_ (mg g^−1^ min^−1/2^) are the rate constants of the pseudo-first-order, pseudo-second-order, and intra-particle diffusion models, respectively. *C* is the intercept related to the boundary layer thickness.^28^

#### 2.2.8 Adsorption isotherm

To describe the equilibrium adsorption behavior and interactions between CR, Cr(vi) molecules, and the MG–AL surface, the Langmuir, Freundlich, and Temkin isotherm models were employed. Their linear forms are as follows:

Langmuir isotherm equation 7
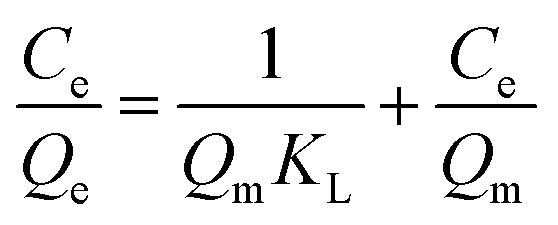


Freundlich isotherm equation 8
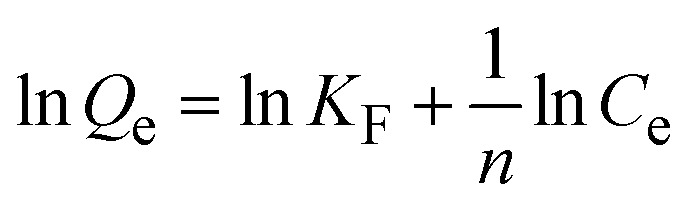


Temkin isotherm equation

9 *Q*_e_ = *B* ln *K*_*t*_ + *B* ln *C*_e_

where *C*_e_ (mg L^−1^) represents the equilibrium concentration of CR and Cr(vi), *Q*_m_ (mg g^−1^)signifies the maximum adsorption capacity of CR and Cr(vi) adsorbed onto the MG–AL adsorbents at equilibrium, *K*_L_ (L mg^−1^) is the Langmuir constant, *K*_F_ (mg g^−1^). (L mg^−1^)^1/*n*^)], *K*_*t*_ is the equilibrium binding constant, *n* reflects adsorption intensity in the Freundlich model, *B* = *RT*/*b* is related to the adsorption heat in the Temkin model, where *R* is the gas constant, and *T* is the absolute temperature.^29,30^

#### 2.2.9 Reusability

The reusability of the MG–AL composite was assessed through an adsorption–desorption–regeneration cycle using hydrochloric acid as the desorbing agent. After adsorption of CR dye and Cr(vi) ions, desorption was carried out by treating the spent adsorbent with HCl solutions (0.1–1.0 M) under continuous stirring for 1 h, effectively recovering the bound contaminants. The regenerated material was then thoroughly washed with deionized water until neutral pH was achieved to eliminate residual acidity, followed by drying at 60 °C to restore its original adsorption capacity.^29^

## 3. Results and discussion

### 3.1 FT-IR analysis

**Fig. 2:**
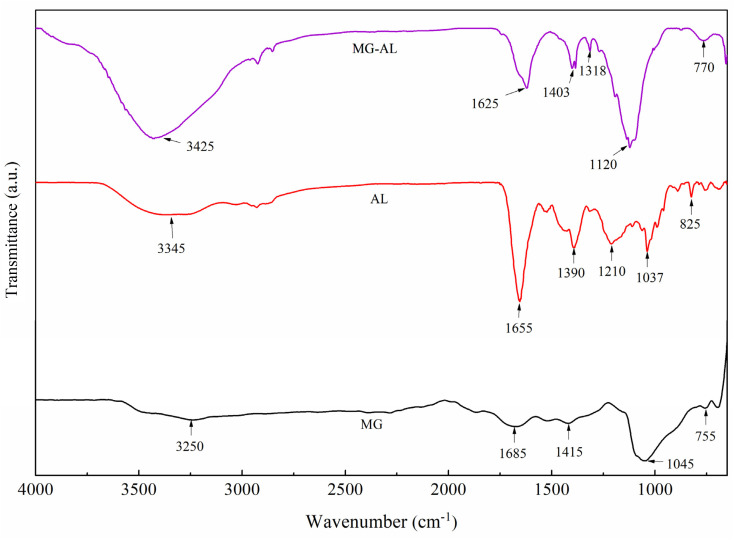
FT-IR spectra of MG, AL, and MG–AL composite.

Fig. 2 reveals the FT-IR spectra of MG, AL, and MG–AL composite. For MG, the spectrum shows O–H stretching vibration at 3250 cm^−1^, indicative of hydroxyl groups and adsorbed moisture on defect sites, along with a prominent C=C stretching band at 1685 cm^−1^ that is characteristic of sp^2^ hybridized graphitic domains. Additional bands at 1415, 1045, and 755 cm^−1^ correspond to C–H bending, C–O stretching, and aromatic C–H deformation. In contrast, AL displays a broad O–H/N–H stretching band at 3345 cm^−1^, a strong carbonyl absorption at 1655 cm^−1^, and several distinct peaks at 1390, 1210, 1037, and 825 cm^−1^, attributed to C–N stretching, C–O vibrations, and aromatic skeletal modes, consistent with its aminated lignin framework. Upon composite formation, the MG–AL spectrum exhibits distinct shifts and alternations in peak intensities compared to those of its components, including strong intermolecular interactions. Specifically, the broad peak observed around 3200–3500 cm^−1^, attributed to O–H and N–H stretching vibrations in both MG and AL, becomes broader shifts in the MG–AL composite, suggesting hydrogen bond formation between AL hydroxyl/amine groups and graphene surface oxygen functionalities.^6^ The C=C/C=O region shows a downshift to 1625 cm^−1^, indicating conjugation/interaction between the graphene π-system and AL carbonyls (electronic coupling and possible π–π stacking or charge transfer).^31^ Likewise, strengthened absorptions at 1403 and 1318 cm^−1^ reflect C–N related vibrations and modified aromatic ring modes attributed to chemical/physical linkage of MG and AL.^32^ Additionally, the band at 1120 cm^−1^ (C–O–C/C–O stretching) and the aromatic deformation at 770 cm^−1^ further confirm retention of lignin structures within the composite.^33^ These spectral changes confirm the successful formation of the MG–AL composite through synergistic interactions, supporting the functionalization of graphene with bio-derived polymers.

### 3.2 SEM analysis

The SEM images of MG, AL, and MG–AL composite offer an in-depth look at the structural changes that occur when these materials are combined.

**Fig. 3:**
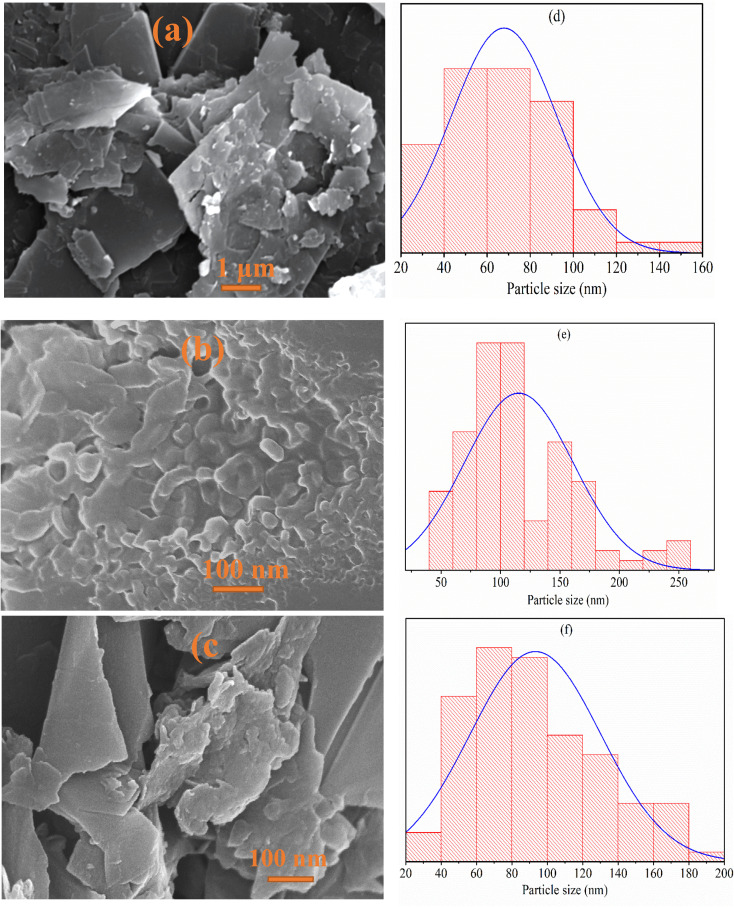
SEM image of (a) MG, (b) AL, and (c) MG–AL composite and histogram graph of (d) MG, (e) AL, and (f) MG-AL composite.

The SEM micrographs and particle size distribution histograms clearly demonstrate the morphological evolution and size variation of MG, AL, and their composite MG–AL. As shown in Fig. 3(a and d), MG exhibits a sheet-like structure with wrinkled surfaces and sharp edges, typical of exfoliated graphene, with an average particle size centered around 67 nm.^34^ In contrast, Fig. 3(b and e) reveals that AL possesses a rough, irregular, and sponge-like morphology with abundant voids and cavities, confirming its highly porous nature. This porosity originates from the disruption of the native lignin structure upon amination, where the introduction of amine groups increases intermolecular spacing and generates a disordered framework. The porous structure enhances surface area and functional group accessibility, reflected in its broader particle size distribution centered around 115 nm.^35^ Upon composite formation, Fig. 3(c and f), MG–AL displays a more compact, interconnected, and heterogeneous structure where AL is uniformly anchored onto MG sheets, reducing agglomeration and promoting interfacial contact. The particle size distribution of MG–AL (93 nm average) lies between MG and AL, reflecting the synergistic integration of both components. This morphological tailoring and size modulation confirm the successful fabrication of the MG–AL hybrid, endowing it with improved dispersion stability and enhanced interfacial properties suitable for advanced adsorption and functional applications.^36^

### 3.3 TGA and DTG analysis

The thermogravimetric and derivative thermogravimetric (DTG) analysis of MG, AL, and MG–AL composite provides important insights into the thermal stability and degradation patterns of these substances, emphasizing the synergistic effects of combining graphene with aminated lignin.

MG demonstrates remarkable thermal stability, with minimal weight loss until temperatures exceed 600 °C, reflecting the robustness of graphene's carbon structure. The overall mass loss for MG up to 800 °C was around 26.25%, suggesting the presence of thermally stable carbonaceous structures.^37^ In contrast, AL showed a distinct multi-step degradation pattern. An initial weight reduction of 19.58% below roughly 200 °C is associated with moisture release and the loss of low-molecular-weight volatiles. The second and most significant decomposition phase occurred between 200 and 600 °C, with a 36.92% mass loss, attributed to the thermal breakdown of lignin's complex phenolic network, including the cleavage of ether linkages and aromatic rings and the decomposition of introduced amino functional groups. An additional 43.42% weight loss was noted beyond 800 °C, indicating the extensive degradation of the carbon backbone. The substantial total weight loss highlights AL's relatively poor thermal stability when isolated.^38^ The MG–AL composite displayed synergistic thermal behavior. An initial weight reduction of 5.82% occurred below 200 °C due to moisture desorption and light volatiles, which is significantly lower than that of pure AL, indicating improved water resistance. Between 200 and 600 °C, a 7.32% weight loss was observed, reflecting the partial decomposition of the lignin structure. This reduced loss compared to AL suggests that the incorporation of MG effectively stabilizes the composite, possibly through π–π stacking, hydrogen bonding, or electrostatic interactions between the MG sheets and aminated lignin chains. The total weight loss of the composite up to 800 °C is approximately 39.91%, which is much lower than that of AL and comparable to or slightly, if not higher than, that of MG, confirming the enhanced thermal stability.^39^

**Fig. 4:**
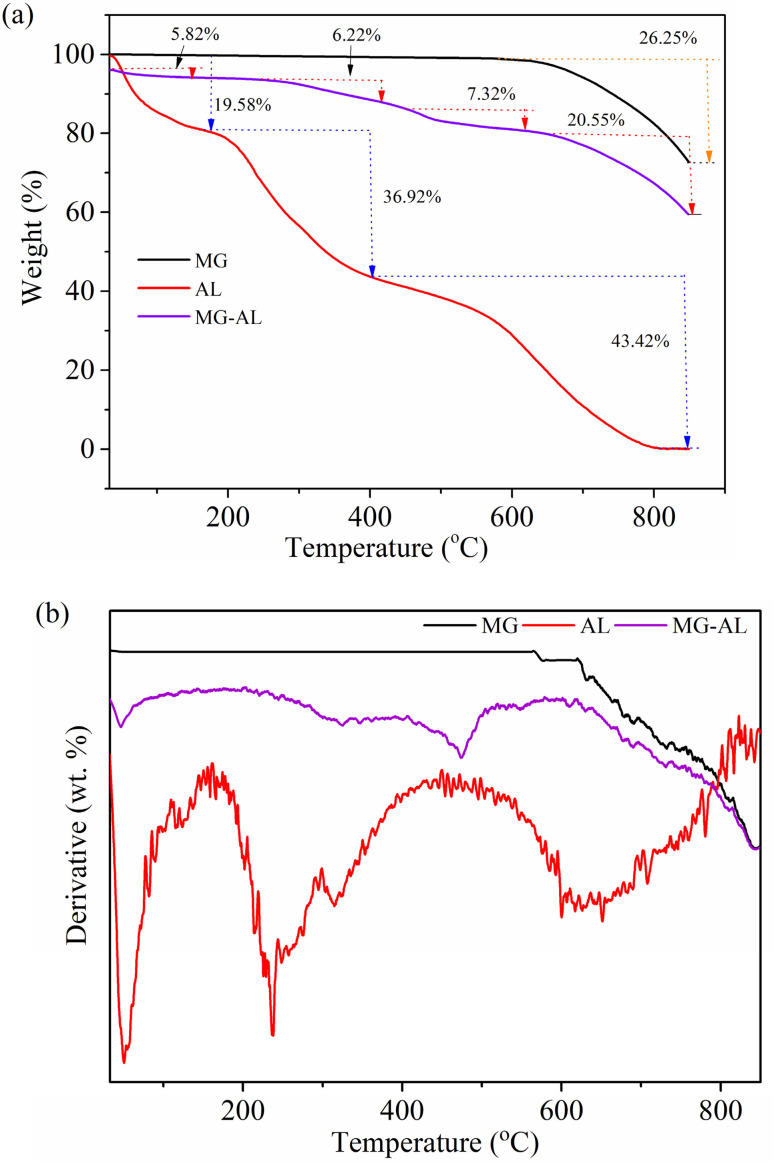
(a) TGA and (b) DTG spectra of MG, AL, and MG–AL composite.

The DTG profiles (Fig. 4b) provide deeper insights into the degradation dynamics of these components. AL exhibits multiple sharp decomposition peaks, corresponding to the stepwise breakdown of its amorphous backbone, dehydration, and cleavage of oxygenated and amine-containing groups, reflecting its poor thermal stability. In contrast, MG shows a nearly flat baseline with only a slight mass-loss, characteristic of its graphitic nature and exceptional structural integrity. The MG–AL composite reveals broadened and suppressed degradation peaks compared to AL, indicating that the incorporation of MG sheets effectively stabilizes the AL matrix by restricting chain mobility and slowing thermal scission.

### 3.4 XRD analysis

**Fig. 5:**
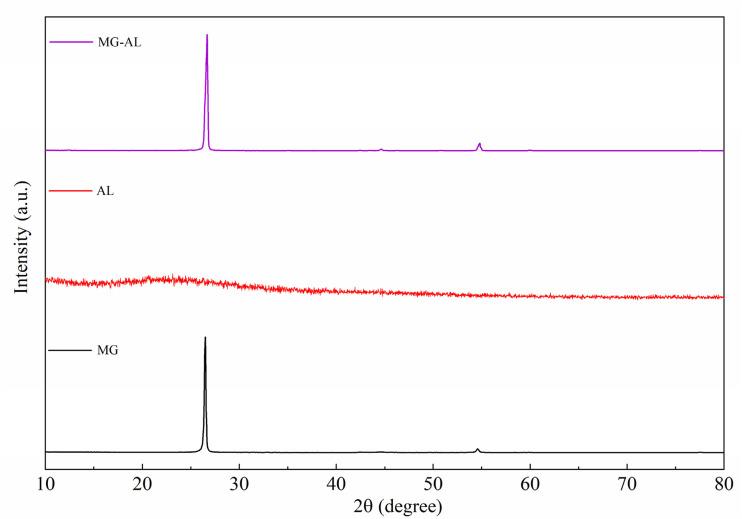
XRD spectra of MG, AL, and MG–AL.

XRD was used to study the structures and crystallinities of MG, AL, and the MG–AL composite, and the findings are shown in Fig. 5.

The typical XRD pattern for graphene is characterized by a distinct peak near 2*θ* = 26.5^°^, which corresponds to the (002) plane and reflects its layered graphitic structure.^40^ The crystallinity index of MG was calculated to be 60.61%, reflecting the partially ordered nature of the graphitic domains. In contrast, AL displays a broad, amorphous halo centered around 2*θ* = 20−25^°^, indicative of its highly disordered polymeric nature.^41^ The absence of long-range ordering in AL can be attributed to the presence of multiple functional groups and a disordered lignin backbone, which inhibit crystallite formation. Interestingly, the MG–AL composite displays a pronounced peak at 2*θ* = 26.7^°^, similar to MG, along with minor reflections at higher angles. This indicates that the crystalline framework of MG is preserved even after hybridization with AL.^42^ Moreover, the crystallinity index of the composite was enhanced to 67%, which is higher than that of MG. This increase suggests that the interaction between the MG layers and AL matrix restricts the random restacking of MG sheets, thereby improving the degree of structural ordering. The improved crystallinity also indicates the presence of strong interfacial bonding and electrostatic interactions between the oxygenated/aminated groups of AL and the MG sheets. Such interfacial interactions not only stabilize the composite structure but also enhance its thermal and chemical durability, which is advantageous for adsorption, electrochemical, and catalytic applications.

### 3.5 Adsorption analysis

#### 3.5.1 Effect of initial concentration and contact time

**Fig. 6:**
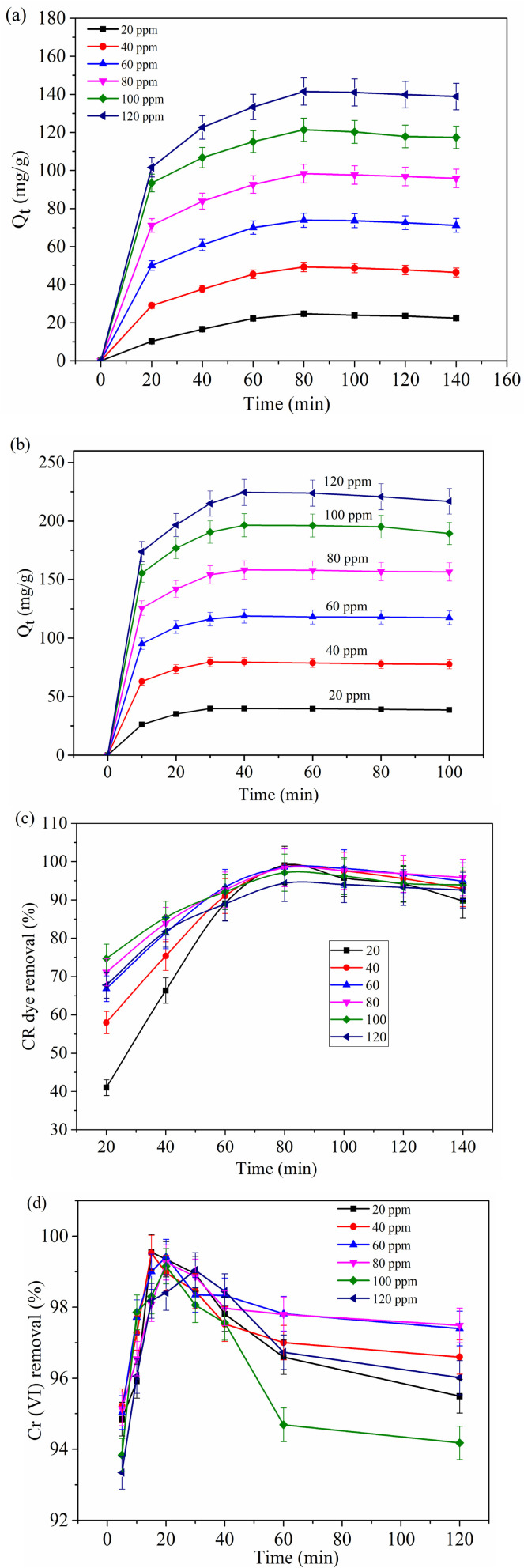
Effect of initial concentration and contact time on adsorption capacity (*Q*_t_) of (a) CR dye, and (b) Cr(vi) on removal efficiency of (c) CR dye and (d) Cr(vi).

Fig. 6 illustrates the effect of initial concentration and contact time on the adsorption performance of the MG–AL adsorbent toward CR dye and Cr(vi), presented in terms of adsorption capacity (*Q*_t_) and removal efficiency.

The adsorption curve for the two types of pollutants reveals two particular stages. Fig. 6 shows, as the initial concentration increases, the adsorption capacity rises because more pollutant molecules are available to occupy the active sites. However, the removal percentage does not increase proportionally, since the fixed amount of adsorbent cannot capture all molecules at higher concentrations, resulting in a lower fraction of total pollutant removed. Initially, the adsorption capacity increases gradually as the contact time progresses, followed by a subsequent phase where the capacity stabilizes and remains nearly constant. This pattern is likely due to the diminishing availability of active adsorption sites, which become increasingly occupied by pollutant ions over time.^43^

Both CR dye and Cr(vi) show a rapid initial increase in adsorption capacity over time, eventually reaching a plateau that signifies equilibrium. Nonetheless, the time to reach equilibrium and the maximum capacities vary between these two pollutants. For CR dye (Fig. 6a), the adsorption capacity consistently increases with concentration, achieving approximately 121.3 mg g^−1^ at 100 ppm after about 80 minutes. In contrast, Cr(vi) (Fig. 6b) attains a much higher adsorption capacity of around 196.4 mg g^−1^ at the same concentration, with equilibrium being reached much more swiftly within 40 minutes, indicating stronger or more favorable interactions with the adsorbent surface.^44^ Regarding removal efficiency, both CR dye and Cr(vi) achieve 97.1% and 98.2% removal, respectively. However, CR dye exhibits a gradual increase in removal efficiency over time, especially at lower concentrations (*e.g.*, 20 ppm reaches about 99.05% after 80 minutes).^17^ On the other hand, Cr(vi) demonstrates a more pronounced initial increase and rapid achievement of peak efficiency, although a slight decline is noted at higher concentrations after equilibrium, possibly due to desorption or competitive interactions. These differences might be due to the molecular structure, size, and charge of the adsorbates. Cr(vi), often present as chromate or dichromate ions, may have a higher affinity for functional groups on the adsorbent surface compared to the larger CR dye molecules, resulting in faster kinetics and greater adsorption capacity.^45^

#### 3.5.2 Effect of adsorbent dose

**Fig. 7:**
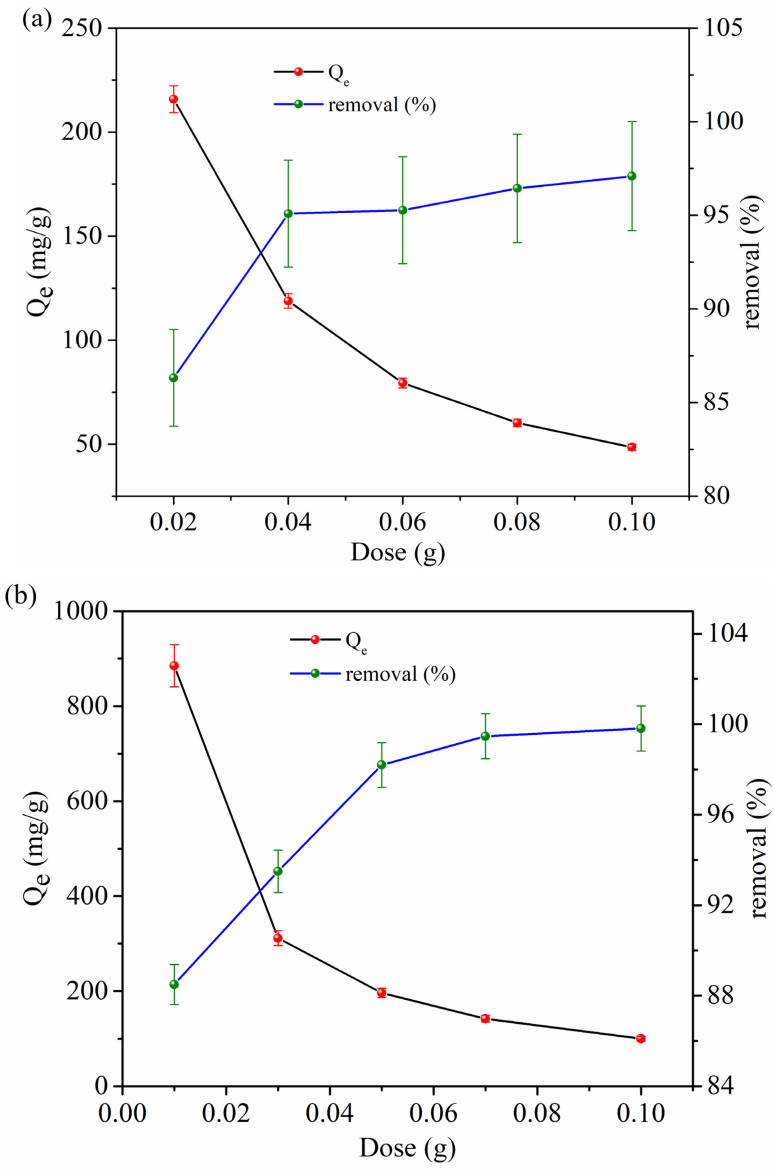
Effect of dose on equilibrium adsorption capacity (*Q*_e_) and removal percentage (a) CR dye, and (b) Cr(vi).

The influence of adsorbent dosage on the equilibrium adsorption capacity (*Q*_e_) and removal efficiency for CR dye and Cr(vi) is presented in Fig. 7.

There is a noticeable inverse correlation between *Q*_e_ and the adsorbent dosage for both contaminants, whereas the removal efficiency consistently rises with increasing dosages. As shown in Fig. 7a, the *Q*_e_ for CR dye significantly drops from 215.8 mg g^−1^ to 48.54 mg g^−1^ as the dosage rises from 0.02 g to 0.10 g. This decline in *Q*_e_ is due to the excess of active sites compared to the constant number of dye molecules in the solution, leading to less efficient use per unit mass of the adsorbent. In addition, particle aggregation at higher dosages can reduce the effective surface area and hinder intraparticle diffusion, further decreasing the *Q*_e_ values. Nevertheless, the removal efficiency steadily improves, reaching 97.09% at higher dosages, due to the greater availability of binding sites for capturing pollutants.^21,46^ A similar pattern is observed for Cr(vi) in Fig. 7b, where *Q*_e_ decreases sharply from 885 mg g^−1^ at 0.01 g to 99.81 mg g^−1^ at 0.10 g. It is noteworthy that the initial *Q*_e_ values for Cr(vi) are much higher than those for CR dye at all dosages, highlighting the stronger binding affinity and potentially more favorable electrostatic interactions of Cr(vi) species with the adsorbent. The removal efficiency also increases with increasing dosage, and around 88.5–99.81%, indicating effective sequestration of Cr(vi) even at moderate dosages.^7^ The comparative analysis shows that although both pollutants follow similar dose-dependent trends, Cr(vi) exhibits a significantly higher adsorption capacity, likely due to its smaller hydrated ionic radius, higher charge density, and stronger affinity for functional groups on the adsorbent surface.

#### 3.5.3 Effect of pH

**Fig. 8:**
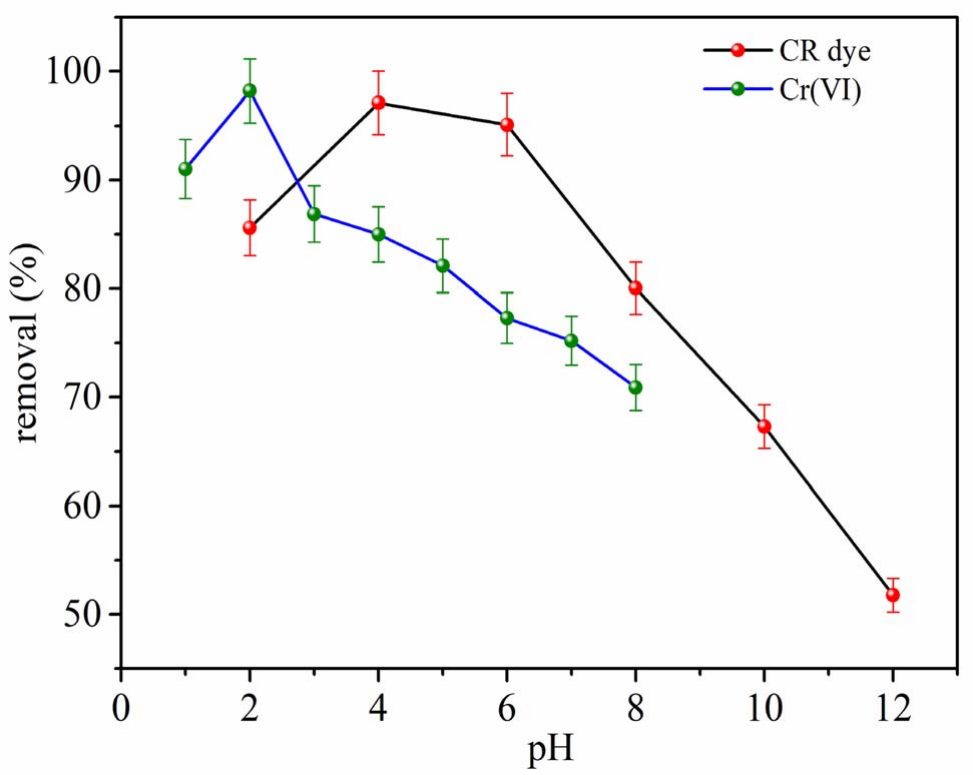
Effect of pH on removal efficiency of CR dye (red dots, black line), and Cr(vi) (green dots, blue line).

The effect of solution pH on the adsorption performance of the MG–AL adsorbent toward CR dye and Cr(vi) is depicted in Fig. 8. The pH of the solutions was adjusted by the addition of 0.1 mol L^−1^ HCL and NaOH.

The adsorption capacity (*Q*_e_) and removal percentage are highly dependent on pH, highlighting the importance of surface charge, pollutant speciation, and electrostatic interactions in the adsorption process. As depicted in Fig. 8, the adsorption of CR dye is particularly sensitive to pH, achieving optimal results at pH 4, where *Q*_e_ reaches approximately 121.3 mg g^−1^ and removal efficiency surpasses 97.1%. This optimal adsorption performance under mildly acidic conditions is due to electrostatic attractions between the protonated amine groups (–NH_3_^+^) on the MG–AL surface and the anionic sulfonate groups (–SO_3_^−^) of CR, resulting in strong Coulombic interactions.^47^ Beyond pH 6, both *Q*_e_ and removal percentage show a significant decline. At pH 8, removal efficiency falls to 80.04%, eventually reaching 51.77%, respectively, at pH 12. This reduction is mainly due to the deprotonation of amine groups on MG–AL at higher pH levels, which reduces the positive surface charge and causes electrostatic repulsion between the negatively charged adsorbent surface and anionic dye species. Additionally, competition from hydroxide ions for active sites may hinder dye adsorption.^48^

Conversely, the adsorption pattern of Cr(vi) (Fig. 8) peaks at pH 2, with *Q*_e_ and removal efficiency reaching around 196.4 mg g^−1^ and 98.2%, respectively. This aligns with the known speciation of Cr(vi) in aqueous solutions, where CrO_4_^−^, Cr_2_O_7_^2−^, and HCrO_4_^−^ are the predominant species under highly acidic conditions. At low pH, the adsorbent surface is positively charged, facilitating strong electrostatic interactions with anionic Cr(vi) species. At pH 8, removal efficiency drops to 70.9%. As pH increases, adsorption performance gradually diminishes due to reduced electrostatic attraction and increased competition from OH^−^ ions, which hinder Cr(vi) binding.^49^

#### 3.5.4 Effect of temperature

**Fig. 9:**
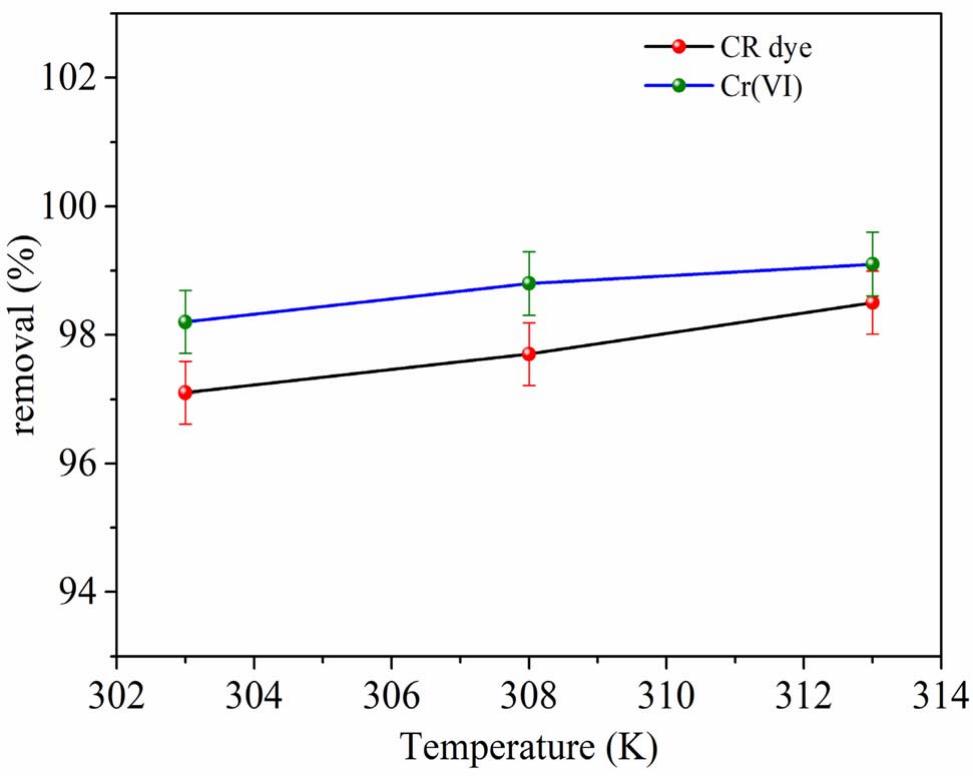
Effect of temperature on removal efficiency of CR dye (red dots, black line), and Cr(vi) (green dots, blue line).

The influence of temperature on the adsorption behavior of CR dye and Cr(vi) was investigated in the temperature range between 303–313 K, as illustrated in Fig. 9.

The adsorption capacity for CR dye gradually rises from approximately 121.3 mg g^−1^ at 303 K to about 123.12 mg g^−1^ at 313 K. Similarly, the removal efficiency slightly improves from 97.1% to 98.5% within the same temperature range. The enhanced adsorption performance at higher temperatures can be attributed to the increased kinetic energy of dye molecules, which promotes faster diffusion from the bulk solution to the adsorbent surface. In addition, elevated temperatures reduce the viscosity of the solution and enhance the accessibility of active sites, facilitating stronger interactions between the dye molecules and the adsorbent surface.^46^

A similar trend was observed for Cr(vi) adsorption (Fig. 9), where *Q*_e_ increased from around 196.4 mg g^−1^ at 303 K to 198.2 mg g^−1^ at 313 K, and the removal efficiency rose from 98.2% to 99.1%. The slight improvement with temperature suggests that higher thermal energy enhances ion mobility and promotes more effective electrostatic interaction and surface binding between Cr(vi) ions and the functional groups of the adsorbent.^50^ Thus, increasing temperature favors faster mass transfer and better utilization of the active sites, resulting in improved overall removal efficiency for both contaminants.

#### 3.5.5 Thermodynamics study

**Fig. 10:**
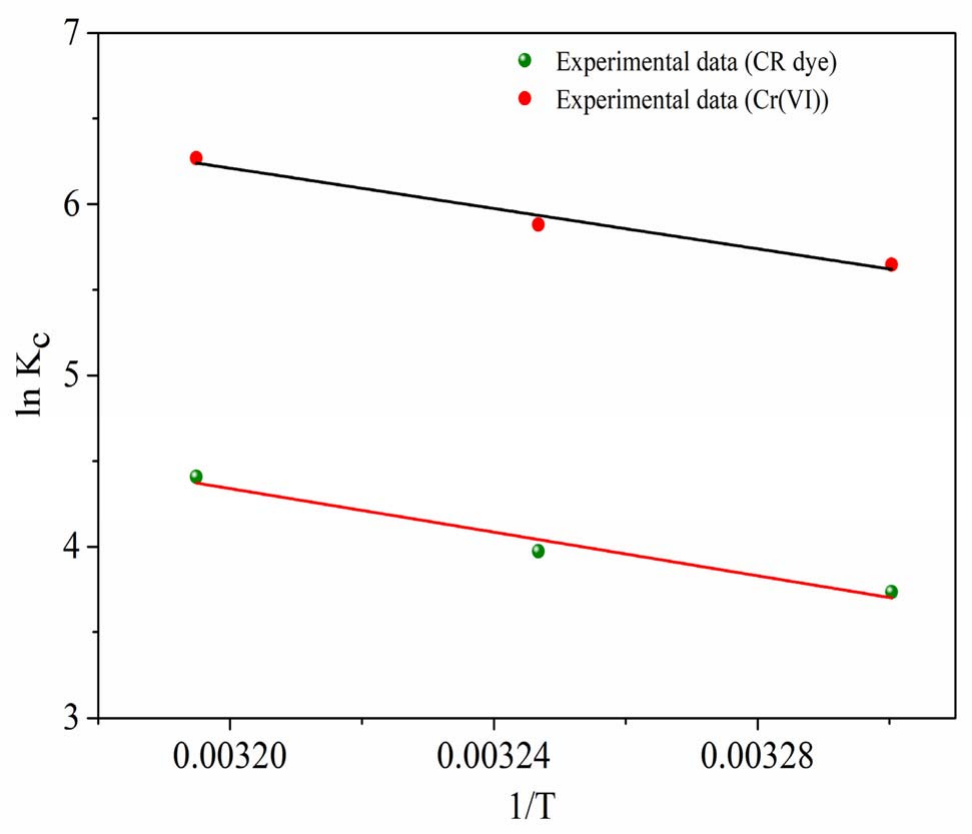
Thermodynamics study of MG–AL composite on CR dye and Cr(vi) adsorption.

A thermodynamic study was conducted to gain a deeper understanding of the nature, adsorption spontaneity, and mechanism of CR dye and Cr(vi) onto the MG–AL composite, as presented in Fig. 10. The values of Δ*G*^0^ as well as the values of standard enthalpy change (Δ*H*^0^) and standard entropy change (Δ*S*^0^) were calculated with the help of Gibbs free energy and van't Hoff equation:

10 Δ*G*^0^ = Δ*H*^0^ − *T*Δ*S*^0^

11
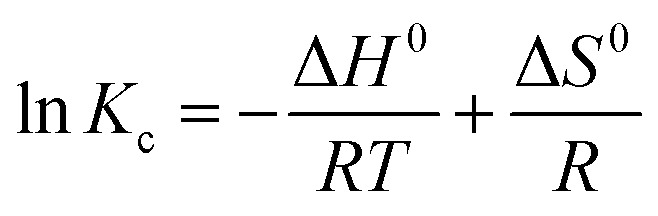


The values of Δ*G*^0^, Δ*H*^0^, and Δ*S*^0^ were calculated as shown in Table 1.

**Table 1:** Thermodynamic parameters obtained for the adsorption process of CR dye and Cr(vi) using MG–AL composite

	Δ*S*^0^ (kJ mol^−1^)	Δ*H*^0^ (kJ mol^−1^)	−Δ*G*^0^ (kJ mol^−1^)		
			303	308	313
Congo red	0.205	53.02	9.09	10.12	11.14
Cr(vi)	0.208	49.08	14.15	15.19	16.24

The positive enthalpy change values (Δ*H*^0^ = 53.02 kJ mol^−1^ for CR dye and 49.09 kJ mol^−1^ for Cr(vi)) indicate that both processes are endothermic, meaning that higher temperatures enhance the adsorption of these pollutants.^51,52^ The positive entropy changes (Δ*S*^0^ = 0.205 and 0.208 kJ mol^−1^ K^−1^ for CR dye and Cr(vi), respectively) imply an increase in disorder at the solid–liquid interface during adsorption, likely due to structural rearrangement and desolvation effects.^50^ Additionally, the Gibbs free energy changes (Δ*G*^0^) for both systems were negative over the temperature range examined (−9.10 to −11.15 kJ mol^−1^ for CR dye and −14.16 to −16.24 kJ mol^−1^ for Cr(vi)), signifying that the adsorption processes are spontaneous and thermodynamically favorable.^53^ Importantly, Cr(vi) showed more negative Δ*G*^0^ values and a greater adsorption capacity than CR dye, indicating a stronger driving force and affinity for the adsorbent, possibly due to its smaller ionic size and stronger electrostatic interactions.

#### 3.5.6 Adsorption kinetics

**Fig. 11:**
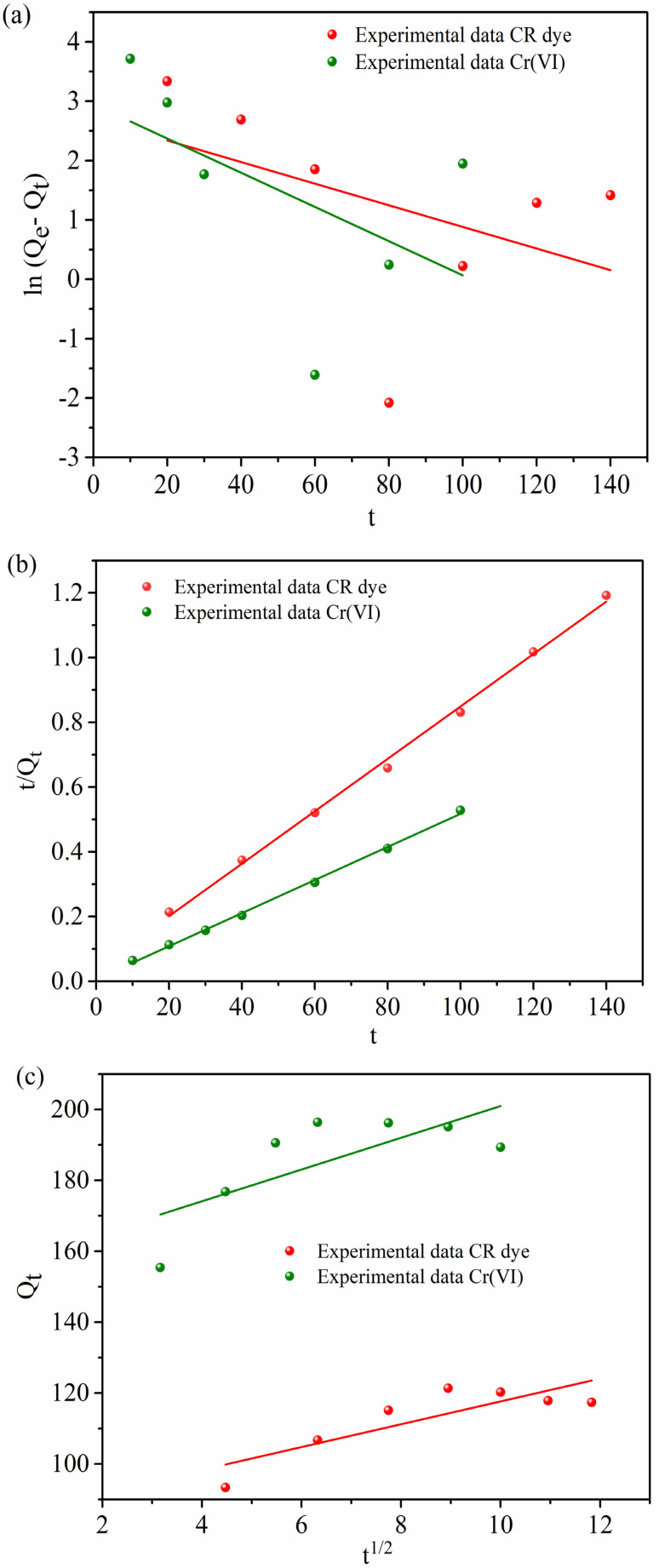
Kinetic model for Congo Red dye and Cr(vi) adsorption by MG–AL composite (a) Pseudo 1st order (b) Pseudo 2nd order and (c) intra-particular diffusion model.

Fig. 11 illustrates the adsorption kinetics of CR dye and Cr(vi) onto a MG–AL composite, which were assessed using kinetic models: pseudo-first-order, pseudo-second-order, and intraparticle diffusion models, and the parameter values are summarized in Table 2.

**Table 2:** Different kinetic parameters obtained for the adsorption of CR dye and Cr(vi) on MG–AL composite

Kinetics		CR dye	Cr(vi)
Pseudo 1st order	*Q*_e_ (mg g^−1^)	14.93	13.14
	*K*_1_ (min^−1^)	0.00015	0.00026
	*R* ^2^	0.19	0.28
	*Q*_exp_ (mg g^−1^)	121.3	196.4
	Error (%)	87.69%	93.3%
Pseudo 2nd order	*Q*_e_ (mg g^−1^)	123.45	196.07
	*K*_2_ (g mg^−1^.min)	0.0016	0.0041
	*R* ^2^	0.99	0.99
	Error (%)	1.77%	0.16%
Intra-particle diffusion	*K*_diff_	3.21	53.1
	*C*	85.43	172.17
	*R* ^2^	0.71	0.53

The pseudo-first-order kinetic model (Fig. 11a) exhibited poor conformity with the experimental data, as evidenced by low correlation coefficients (*R*^2^ = 0.19 for CR and 0.28 for Cr(vi)) and large discrepancies between calculated and experimental adsorption capacities, resulting in errors exceeding 87%. This indicates that the adsorption rate could not be adequately described by a physisorption-controlled mechanism.^39,54^

In contrast, the pseudo-second-order kinetic model (Fig. 11b) provided an excellent fit to the experimental data, yielding correlation coefficients of 0.99 for both adsorbates and negligible errors (1.77% for CR and 0.16% for Cr(vi)). Moreover, the calculated equilibrium capacities (*Q*_e, cal_ = 123.45 mg g^−1^ for CR and 196.07 for Cr(vi)) were in close agreement with the experimental values (*Q*_e exp_ = 121.3 and 196.4 mg g^−1^, respectively). These observations strongly suggest that the adsorption rate is governed by chemisorptive interactions, where electron exchange or sharing occurs between the functional groups of the adsorbent and the adsorbate species.^55,56^

Additional insights into the adsorption mechanism were gained from the intraparticle diffusion model (Fig. 11c). The multilinear plot indicates that while intraparticle diffusion plays a role, it is not the only rate-controlling step.^7,57^ The considerable intercept values (C = 85.43 and 172.17 mg g^−1^) further indicate the presence of a pronounced boundary-layer effect, implying that film diffusion dominates during the initial adsorption stage, followed by gradual pore diffusion as equilibrium is approached.

#### 3.5.7 Adsorption isotherm study

**Fig. 12:**
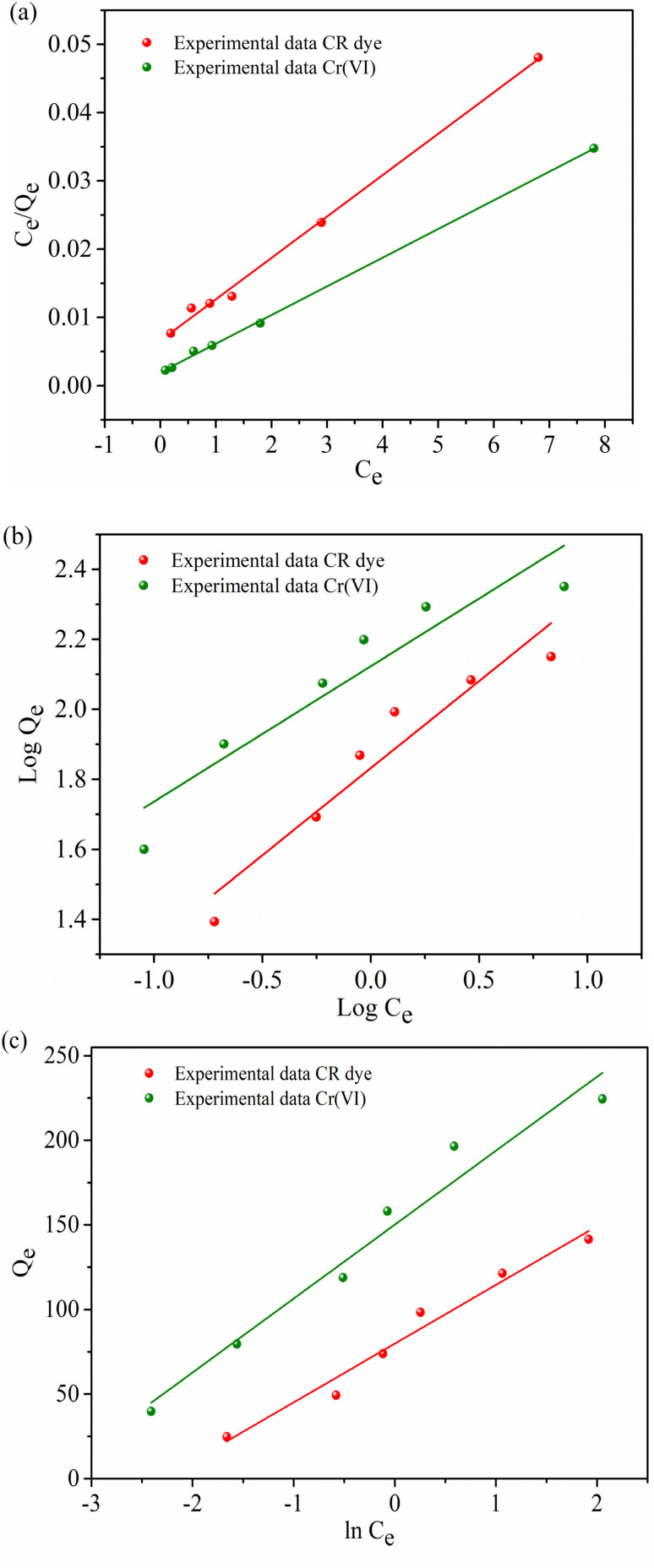
Adsorption Isotherm of CR dye and Cr(vi) from aqueous solution by MG–AL composite, (a) Langmuir isotherm, (b) Freundlich isotherm, and (c) Temkin isotherm.

The efficiency of the MG–AL composite for CR dye and Cr(vi) is evaluated using the adsorption isotherm model. Experimental data were fitted with the Langmuir, Freundlich, and Temkin isotherm models, and the corresponding plots are shown in Fig. 12. Table 3 displays the parameters obtained from fitting these models to the experimental data.

**Table 3:** Different isotherm parameters obtained for the adsorption of CR dye and Cr(vi) on MG–AL composite

Isotherm		CR dye	Cr(vi)
Langmuir	*Q*_exp_ (mg g^−1^)	121.3	196.4
	*Q*_m_ (mg g^−1^)	149.25	238.09
	*K*_L_ (L mg^−1^)	3.80	2.1
	*R* ^2^	0.96	0.99
	Error (%)	18.72%	17.51%
Freundlich	*n*	1.34	2.59
	*K*_F_ (mg g^−1^)	5.009	8.35
	*R* ^2^	0.90	0.86
Temkin	*B*_t_ (J mol^−1^)	34.71	43.69
	*K*_t_ (L mg^−1^)	2.29	3.43
	*R* ^2^	0.96	0.95

Among these, the Langmuir model provided the best correlation with the experimental data, exhibiting high regression coefficients (*R*^2^ = 0.96 for CR and 0.99 for Cr(vi)) and low relative errors (18.72% and 17.51%, respectively). The close agreement between the experimental (*Q*_e_,_exp_ = 121.3 and 196.4 mg g^−1^) and theoretical maximum adsorption capacities (*Q*_m_ = 149.25 and 238.09 mg g) confirms the applicability of the Langmuir model, indicating that the adsorption process proceeds through monolayer coverage on a homogeneous surface with energetically uniform active sites.^44,56^ In contrast, the Freundlich model showed slightly lower correlation coefficients (*R*^2^ = 0.90 for CR and 0.86 for Cr(vi)), suggesting a less significant contribution from multilayer adsorption. However, the values of the Freundlich constant (*n* = 1.34 for CR and 2.59 for Cr(vi)) being greater than 1 indicate a favorable adsorption process and strong affinity of the adsorbent surface toward both contaminants.^43,53^ The Temkin isotherm also exhibited a good linear fit (*R*^2^ = 0.96 for CR and 0.95 for Cr(vi)), with the Temkin constant (*B*_t_ = 34.71 and 43.69 J mol^−1^) reflecting moderate interactions between the adsorbent and adsorbate. This suggests that a uniform distribution of the heat of adsorption across the MG–AL composite surface and adsorption energy decreases gradually with surface coverage, aligning with the chemisorptive nature of the process inferred from kinetic analysis.^58,59^

#### 3.5.8 UV-Vis analysis for the reduction of Cr(vi) to Cr(iii)

**Fig. 13:**
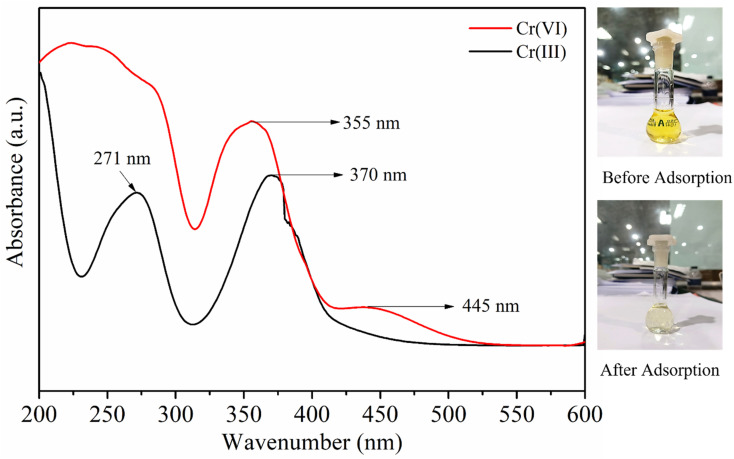
UV-Vis spectra of initial Cr(vi) concentration and after adsorption Cr(vi) concentration.

The UV-Vis absorption spectra of Cr(vi) and Cr(iii) solutions are presented in Fig. 13. The Cr(vi) spectrum exhibits two prominent absorption peaks at 355 nm and 445 nm, which are attributed to the O → Cr^6+^ charge–transfer transitions in chromate (CrO_4_^2−^) and dichromate (Cr_2_O_7_^2−^) species. After adsorption by the MG–AL adsorbent, these characteristic bands significantly decreased in intensity, accompanied by the appearance of new peaks at around 271 nm and 370 nm, which correspond to the ligand-to-metal charge transfer and d–d transitions of Cr^3+^ ions, respectively. The simultaneous attenuation of the Cr(vi) peaks and the emergence of Cr(iii)-related bands clearly indicate the partial reduction of Cr(vi) to Cr(iii) during the adsorption process. This transformation suggests that the adsorption mechanism involves not only electrostatic interaction between Cr(vi) species and the functional groups on the composite surface but also redox reactions, where oxygen-containing groups (*e.g.*, –OH, –COOH) act as electron donors to reduce toxic Cr(vi) to the less toxic Cr(iii). Similar spectral transitions confirming Cr(vi) reduction have been reported in earlier studies.^60,61^

#### 3.5.9 Reusability

**Fig. 14:**
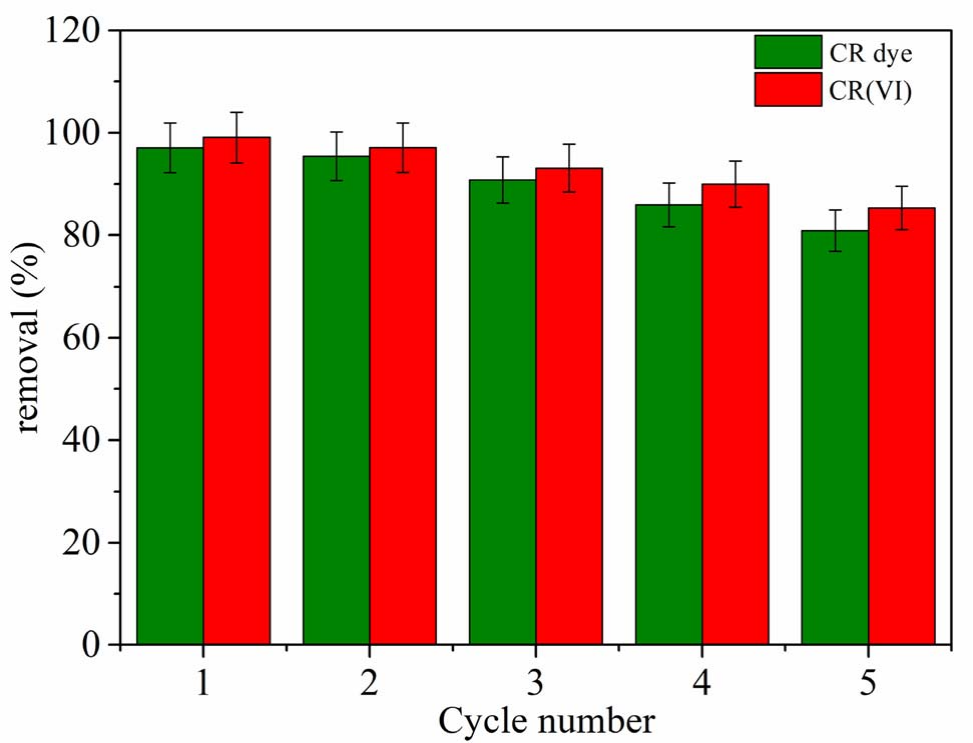
Reusability of MG–AL composite on CR dye, and Cr(vi) adsorption from aqueous solution.

Fig. 14 reveals the potential for reusing the MG–AL composite in adsorbing CR dye and Cr(vi) from water was thoroughly evaluated over five sequential adsorption–desorption cycles to determine its ability to regenerate and remain stable in operation. The reusability profile revealed that the initial removal efficiencies for both CR dye and Cr(vi) were above 95%, indicating the composite's high surface reactivity and the plentiful presence of active functional sites.^62^ Interestingly, Cr(vi) consistently showed slightly better removal efficiencies than CR dye throughout all cycles, suggesting stronger electrostatic or coordination interactions between Cr(vi) species and the functional groups on the MG–AL composite surface. A gradual but controlled decrease in removal performance was noted over the cycles. By the fifth cycle, the removal efficiency dropped to about 80.89% for CR dye and 85.31% for Cr(vi), indicating only a moderate reduction in adsorptive capacity. This decline could be due to potential site saturation, minor structural degradation, or partial pore blockage from residual adsorbate accumulation during regeneration.^63,64^ Notably, the sustained high adsorption efficiency over multiple cycles highlights the composite's exceptional structural resilience and surface regeneration capability.

#### 3.5.10 Adsorption mechanism

**Fig. 15:**
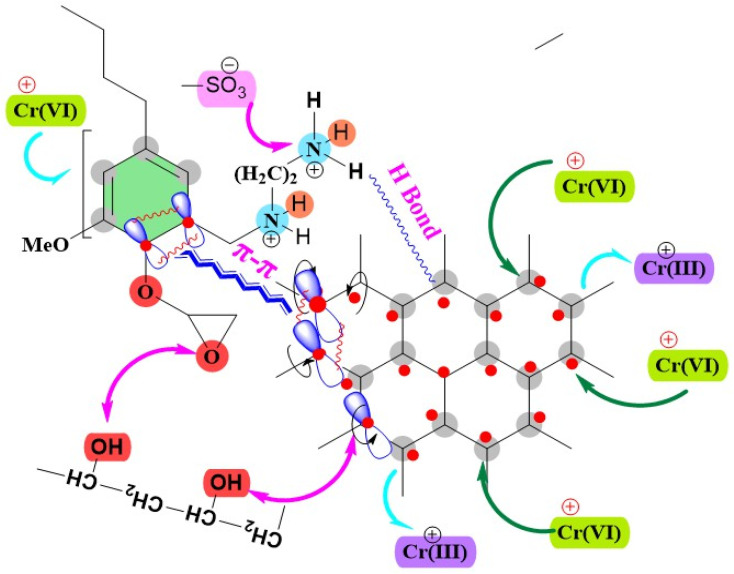
Adsorption mechanism on CR dye and Cr(vi) adsorption by MG–AL composite.

The adsorption processes of CR and Cr(vi) onto the MG–AL composite are influenced by combined physico–chemical interactions facilitated by the composite's specific functionalities (see Fig. 15). For CR, the adsorption is mainly due to the electrostatic attraction between the protonated amine groups (–NH_3_^+^) on AL and the anionic sulfonate groups (–SO_3_^−^) of CR, along with π–π stacking interactions with the sp^2^-hybridized domains of graphene, and hydrogen bonding through surface –OH and –NH_2_ groups.^65,66^ The removal of Cr(vi) in acidic conditions (pH 2) involves the electrostatic attraction of anionic chromate species (*e.g.*, HCrO_4_^−^), partial reduction to Cr(iii) aided by the electron-rich graphene domains, and complexation with amine groups.^67^ Kinetic modeling supports chemisorption for both substances, while Langmuir isotherm fitting suggests monolayer coverage on uniform active sites. The endothermic and spontaneous nature of both processes, as confirmed by thermodynamic parameters, along with improved diffusion and adsorption due to increased porosity and accessibility of functional sites (as shown by SEM, FT-IR, and XRD), emphasizes the effectiveness and reusability of the MG–AL composite.

#### 3.5.11 Limitations of the study and future directions

Although the MG–AL composite exhibited excellent adsorption performance for CR dye and Cr(vi), this study was conducted under batch conditions using synthetic aqueous solutions, which may not fully represent the complexity of real industrial wastewater. Future work should therefore evaluate the composite in real industrial effluents containing competing ions and organic matter. Additionally, while partial reduction of Cr(vi) to Cr(iii) was confirmed, detailed chromium speciation and long-term stability were not investigated and warrant further study using advanced spectroscopic techniques. The adsorption process was limited to laboratory-scale experiments; thus, continuous-flow and fixed-bed column studies are recommended for scale-up. Further optimization of composite formulation, long-term reusability, and sustainability assessment will support practical wastewater treatment applications.

## 4. Conclusion

In this research, a sustainable and effective adsorbent composite was synthesized by combining microwave-exfoliated graphene with aminated lignin sourced from biomass (relevant works and our work are summarized in Table 4). The resulting MG–AL composite showed an exceptional ability to remove both CR dye and Cr(vi) from aqueous solutions. Comprehensive physicochemical analysis confirmed successful functionalization, increased porosity, and enhanced thermal stability. Adsorption tests revealed high capacities of 121.3 mg g^−1^ for CR and 196.4 mg g^−1^ for Cr(vi), with removal efficiencies surpassing 97.1% and 98.2%, respectively, under optimal conditions. Kinetic and isotherm studies suggested a chemisorption-driven process following pseudo-second-order and Langmuir models, while thermodynamic evaluations confirmed the endothermic and spontaneous nature of the adsorption. Notably, the composite maintained significant efficiency over five regeneration cycles, underscoring its potential for repeated use in practical applications. This study highlights the potential of lignin-derived, graphene-enhanced materials for sustainable and scalable wastewater treatment.

**Table 4:** Adsorption of CR dye and Cr(vi) by different adsorbents and their adsorption capacity

Adsorbent	Adsorbate	Adsorption capacity (mg g^−1^)	Reference
EL-PEI@Fe_3_O_4_–Mg	CR dye	74.7	68
Coal graphene	CR dye	129	69
GO-CuFe_2_O_4_	CR dye	37.97	70
Chitosan-GO	CR dye	10.245	71
GO/MgO NCs	CR dye	13.62	72
l-cysteine/rGO/PANI nanocomposite	CR dye	56.57	73
GO-M1, GO-M2 and GO-M3	Cr(vi)	3.5412, 2.3631, and 7.0358	74
α-FeO(OH)/GOCS	Cr(vi)	63.19	75
GO and rGO-ZnO nanocomposite	Cr(vi)	19.49 and 25.45	76
G, AC and GAC	Cr(vi)	6.627, 5.455, and 6.354	77
Chitosan quinoxaline schiff base	Cr(vi)	103.09	78
MG–AL composite	CR dye	121.3	Present work
	Cr(vi)	196.4	

## Author contributions

Md. Masum Billah: research work, writing – original draft, and editing; S.M. Fazle Rabbi: methodology, investigation, data analysis, validation, writing – reviewing and editing; Md. Kamruzzaman: idea generation, conceptualization, investigation, formal analysis, writing – reviewing and editing; Mohammad Amirul Hoque: conceptualization, investigation, methodology, data analysis, writing – reviewing and editing; Riyadh Hossen Bhuiyan: conceptualization, investigation, methodology, data analysis, writing – reviewing and editing; Israt Jahan Nisa: methodology, investigation, writing – reviewing and editing.

## Conflicts of interest

The authors state that they have no financial interests or personal connections that might have influenced the research presented in this paper. We confirm that this manuscript is original and has not been submitted for publication elsewhere. Furthermore, we have no conflicts of interest with any individuals.

## Data Availability

All data supporting the findings of this study are available within the article. Additional raw data or supplementary materials supporting this study are available in Mendeley Data at [DOI: https://doi.org/10.17632/cmpkhvn5zc.1].
